# LRF-SRNet: Large-Scale Super-Resolution Network for Estimating Aircraft Pose on the Airport Surface

**DOI:** 10.3390/s23031248

**Published:** 2023-01-21

**Authors:** Xinyang Yuan, Daoyong Fu, Songchen Han

**Affiliations:** School of Aeronautics and Astronautics, Sichuan University, Chengdu 610065, China

**Keywords:** aircraft pose estimation, super resolution, receptive field

## Abstract

The introduction of various deep neural network architectures has greatly advanced aircraft pose estimation using high-resolution images. However, realistic airport surface monitors typically take low-resolution (LR) images, and the results of the aircraft pose estimation are far from being accurate enough to be considered acceptable because of long-range capture. To fill this gap, we propose a brand-new, end-to-end low-resolution aircraft pose estimate network (LRF-SRNet) to address the problem of estimating the pose of poor-quality airport surface surveillance aircraft images. The method successfully combines the pose estimation method with the super-resolution (SR) technique. Specifically, to reconstruct high-resolution aircraft images, a super-resolution network (SRNet) is created. In addition, an essential component termed the large receptive field block (LRF block) helps estimate the aircraft’s pose. By broadening the neural network’s receptive field, it enables the perception of the aircraft’s structure. Experimental results demonstrate that, on the airport surface surveillance dataset, our method performs significantly better than the most widely used baseline methods, with AP exceeding Baseline and HRNet by 3.1% and 4.5%.

## 1. Introduction

Aircraft pose estimation is a fundamental and challenging vision task that is crucial for many downstream tasks, such as intelligent airport security monitoring [1], decreasing aircraft crashes with airport surfaces [2,3,4], assisting subsequent airport control decisions [5,6], and establishing digital twin airports [7,8].

The six degrees of freedom (6D) of an aircraft, including its three translations (x,y,z) and rotation (α,β,γ) around its three axes, are usually referred to as the aircraft’s pose. However, the altitude (*z*), roll (α), and pitch (β) are meaningless for the aircraft on the airport surface in taxiing or parked state, because their values are zero [9,10]. Thus, the 2D position (x,y) and yaw angle (γ) are used to describe the pose of the aircraft on the airport surface.

**Figure 1:**
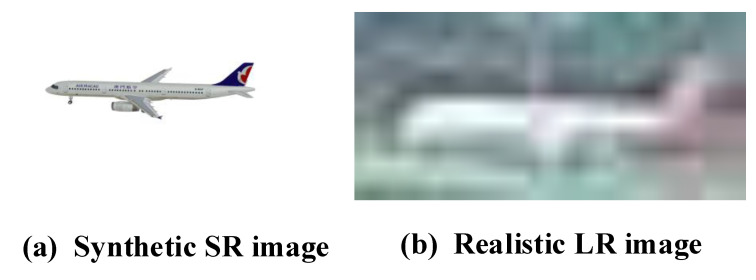
(**a**) Synthetic high-resolution aircraft image of virtual scene and (**b**) low-resolution aircraft image of realistic scene.

The majority of the current methods [10,11,12] focus on studying how to precisely estimate the aircraft pose from high-resolution (HR) aircraft images (e.g., 512×384 or 256×192). Since their synthetic datasets cannot completely simulate realistic and complicated airport field environments, there are massive gaps in the study on estimating aircraft pose in low-resolution (LR) situations (e.g., 128×96), as shown in Figure 1a. Although high-resolution images can provide more details, in real airport scenes, such as long-distance capture, most aircraft images can only be acquired as poor-quality LR images, as shown in Figure 1b. Therefore, it is necessary to use super-resolution methods to help aircraft pose estimation.

**Figure 2:**
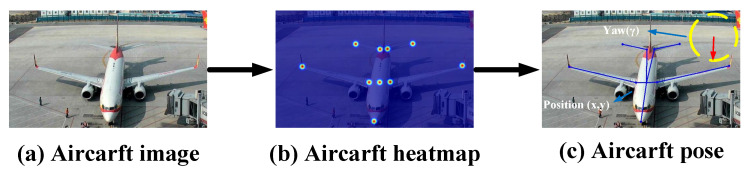
The pose estimation of the aircraft base on the representation of the aircraft skeleton.

The main purpose of the method is to estimate the 2D locations (x,y) of the aircraft joints that make up the aircraft geometry from the image, as illustrated in Figure 2b. The pose of the aircraft must match the geometry of the aircraft. As shown in Figure 2c, the final aircraft pose is represented by the aircraft skeleton, which is made up of the geometrical keypoints of the aircraft and their connections. The majority of deep neural network based pose estimation methods [13,14,15,16,17] currently use the generation of Gaussian heatmaps to represent the positions of actual keypoints, where each pixel in the heatmap indicates the probability of belonging to a particular type of keypoint. This is because it is currently difficult for these methods to directly regress the 2D positions of keypoints on the aircraft surface in the images. Due to the progressive downsampling process’s inevitable loss of resolution, the heatmap’s resolution is typically lower than that of the input image. Because of this, the generated heatmap for a given low-resolution image may be quite small (e.g., 32×24), which causes serious quantization problems when the aircraft keypoint heatmap is recovered to the same size as the input image. As a result, precise aircraft keypoint localization is essential.

In this work, we explore how to deal with the issue of the precise estimation of low-resolution 2D aircraft pose. Super-resolution methods have recently been applied as preprocessing and have significantly improved downstream tasks, such as object detection [18,19,20]. Inspired by this, we extend the SR concept by designing an SRNet as an upstream subnetwork for aircraft pose estimation. Additionally, as the upsampling of SRNet may lead to local texture blurring of the aircraft, we propose a large receptive field block (LRF block) to expand the receptive field to cover the global features of the aircraft. We assess our method using a tough real-world airport surface surveillance dataset that includes images of parked aircraft, inbound and outgoing terminal traffic, and the runway being used for taxiing. We then compare the results to other state-of-the-art baseline methods.

We make the following contributions in this work:

1. The end-to-end low-resolution aircraft 2D pose estimation network LRF-SRNet is proposed, which combines SR methods with pose estimation methods to precisely estimate the pose of an aircraft from low-resolution images.
2. A large receptive field block (LRF block) is created as a core component to assist the network in extending its effective perceptual field and identifying the overall characteristics of the aircraft.
3. The results of our experiments demonstrate that, when applied to the real-world airport surface surveillance dataset, our approach can successfully assist the pose estimate network in improving its performance.

The remainder of the essay is structured as follows. Section 2 looks at related studies. Our particular network model’s structure is presented in Section 3, and the experimental findings are presented in Section 4. A conclusion follows Section 5.

## 2. Related Work

Recent methods for a variety of aircraft vision tasks involving low-resolution images are classified into two categories, depending on the solution ways: adding external super-resolution subnetworks to recover high-quality images and adding internal multiscale perceptual field modules to enhance the network’s performance in extracting aircraft features.

### Super-Resolution-Based Method

Due to the limitations of the imaging equipment and less than ideal air conditions, among other factors, the surveillance video captured by digital imaging payloads in real-world situations is almost always blurry and degraded. As a result, there has been extensive research on how to handle high-resolution reconstruction in low-resolution imaging. He et al. [21] enhanced the spatial image resolution of a video taken by small drones using image fusion technology. Li et al. [22] suggested a technique for combining select high-resolution multispectral remote sensing photos to create low-resolution super-resolution (SR) virtual scenes. The proper detection of tiny blurring airplanes in complicated airport photos is achieved by using an efficient deep belief network (DBN) [23] to rebuild high-resolution features from numerous input images, including grayscale images and two locally thresholded images. By creating high-resolution aircraft from low-resolution remote sensing images, Tang et al. [24] proposed a joint super-resolution and aircraft recognition (Joint-SRARNet) SRARNet to enhance aircraft recognition performance. However, there is still a lack of study on the topic of aircraft pose estimation at low resolution, requiring further research. Thanks to the convolutional receptive field sensing aircraft features, deep convolutional neural network based aircraft pose estimation has recently been a research hot-spot in the field of aviation. The network’s ability to aggregate local features depends on the size and shape of the receptive field, which has a significant impact on the model’s performance. Since images often present complex backgrounds and hazy situations, many studies have looked at how to obtain larger fields of perception at shallower network depths. Zhao et al. [25] suggested a multiscale information augmentation framework (MS-IAF), which accurately identifies multiscale aircraft and their vital parts by stacking perceptual fields of various scale sizes in a multipath way. Li et al. [26] developed a new core component CBL module to increase the receptive field range in the neural network in order to address the issue of aircraft detection in airport field video images that is caused by a long shooting distance, small aircraft targets, and mutual occlusion. Wu et al. [27] enhanced aircraft detection in high-resolution remote sensing images with dense targets and complex backgrounds by improving Mask-rcnn [28] based on atrous convolution. However, the majority of these methods use atrous convolution or multilevel small convolution architectures to implement the large-scale receptive field. Large kernel has recently been proven efficient for effective receptive fields (ERFs) [29] and implements state-of-the-art pure convolutional network architectures on ImageNet classification [30], ADE20K semantic segmentation [31], and COCO target detection [32]. Inspired by this, we create a new core component LRF block to expand the receptive field of contemporary convolutional neural networks for effective capture of aircraft features.

## 3. Methodology

**Figure 3:**
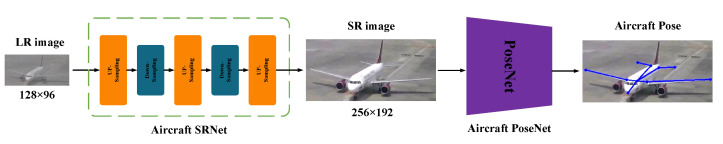
Low-resolution aircraft pose estimation pipline.

To solve the aircraft 2D pose estimation problem at low resolution, as shown in Figure 3, we propose an aircraft super-resolution reconstruction network (Aircraft SRNet) and an aircraft pose estimation network (Aircraft PoseNet). Aircraft SRNet reconstructs the aircraft’s high-resolution information using spaced tandem up- and downsampling blocks. The high-resolution aircraft image is then fed into Aircraft PoseNet as input to predict all of the aircraft’s geometric keypoints and generate the aircraft skeleton pose, as shown in Figure 2.

### 3.1. Aircraft Keypoint Heatmap

**Figure 4:**
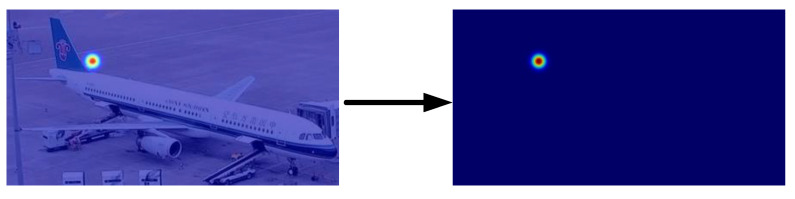
Heatmap of the aircraft’s left tail end point.

To properly represent the actual positions of aircraft geometric keypoints in space, a Gaussian-heatmap-based approach is utilized to describe the positions of aircraft geometric keypoints in the two-dimensional plane as soft annotations. Figure 4 illustrates how a Gaussian kernel with a variance of σ covers the left tail of the aircraft endpoint, and the value of $p_{i}$ at any location in the Gaussian heatmap represents the confidence probability that the endpoint belongs to the left tail of the aircraft. The detailed equation is shown in (1):

(1) $$Hm\left(k\right)=\left\{\begin{array}{cc} \exp\left(-\frac{{∥k-P_{i}∥}_{2}^{2}}{\sigma^{2}}\right) & if\;\exp\left(-\frac{{∥k-P_{i}^{}∥}_{2}^{2}}{\sigma^{2}}\right)>thred\\ 0 & otherwise \end{array}\right.$$

The aircraft’s *k*th geometric keypoint is indicated by the Gaussian heatmap Hm(k) with standard deviation σ. The confidence level is higher for points ($P_{i}$) close to the aircraft’s keypoint and lowers or even reaches zero for locations far from the keypoint when using a threshold (thred) benchmark.

### 3.2. Loss Function

The loss function for our aircraft pose estimation is:

(2) $$L_{pose}=\sum_{t=0}^{t=n}L_{heatmap(k)}$$

$L_{heatmap}$ is the L2loss of the model-predicted aircraft keypoint heatmap. The principal inflection and end points of the aircraft structure are contained in the ten keypoints we choose, including the nose of the aircraft, the left wing tip, the right wing tip, the right horizontal tail tip, the left horizontal tail tip, the point where the left horizontal tail attaches to the fuselage, the point where the right horizontal tail attaches to the fuselage, the point where the left-wing attaches to the fuselage, the point where the right-wing attaches to the fuselage, and the midpoint of the two points. Their distribution preserves the symmetry of the aircraft as a rigid body, making them easily detectable because the relative position relationship between each keypoint is fixed. These n=10 aircraft geometrical keypoint heatmap losses constitute the final aircraft pose estimation losses ($L_{pose}$).

### 3.3. Aircraft Super-Resolution Network

We organize the cross-cascade architecture of upsampling and downsampling blocks [33] in Aircraft SRNet so that the features interact with features between the high-resolution semantic space and the low-resolution semantic space in order to reconstruct the high-resolution features of the aircraft. It reconstructs the high-resolution aircraft features by upsampling blocks before learning the deep semantic features of the aircraft, downprojecting the high-resolution features to low resolution, and then up-projecting the deep semantic features to recover the high-resolution feature maps.

**Figure 5:**
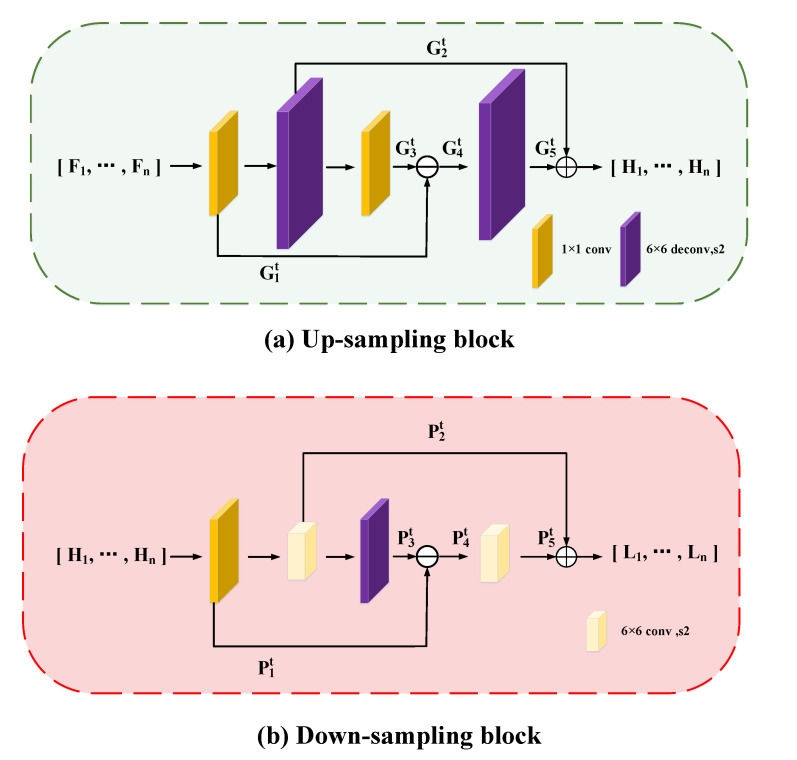
Sampling block in Aircraft SRNet.

Furthermore, Figure 5a depicts the upsampling block’s structural layout. Assuming that the feature input is the feature output tensor from the previous stage $\left[F_{1},\cdots ,F_{n}\right]$, the upsampling block produces a high-resolution feature maps:

(3) $$\left[H_{1},\cdots ,H_{n}\right]=G_{2}^{t}+G_{5}^{t}$$

$G_{5}^{t}$ is the feature map of the final scale-up of the upsampling block, and $G_{2}^{t}$ is the feature tensor of the first scale-up at stage *t*. As shown in Equation (4), a scale-down is carried out in between the two scale-ups, and the reconstructed residuals are propagated forward and self-corrected backward:

(4) $$G_{4}^{t}=G_{1}^{t}-G_{3}^{t}$$

As seen in Figure 5b, using the output feature maps from the previous upsampling $\left[H_{1},\cdots ,H_{n}\right]$, and using the downsampling block, a low-resolution feature maps is produced:

(5) $$\left[L_{1},\cdots ,L_{n}\right]=P_{2}^{t}+P_{5}^{t}$$

$P_{2}^{t}$ is the feature tensor of the first scale-down, and $P_{5}^{t}$ is the feature map of the final scale-down. Following the two scale-downs, a scale-up and residual cascade were carried out, as illustrated in Equation (6):

(6) $$P_{4}^{t}=P_{1}^{t}-P_{3}^{t}$$

### 3.4. Aircraft Pose Estimation Network

**Figure 6:**
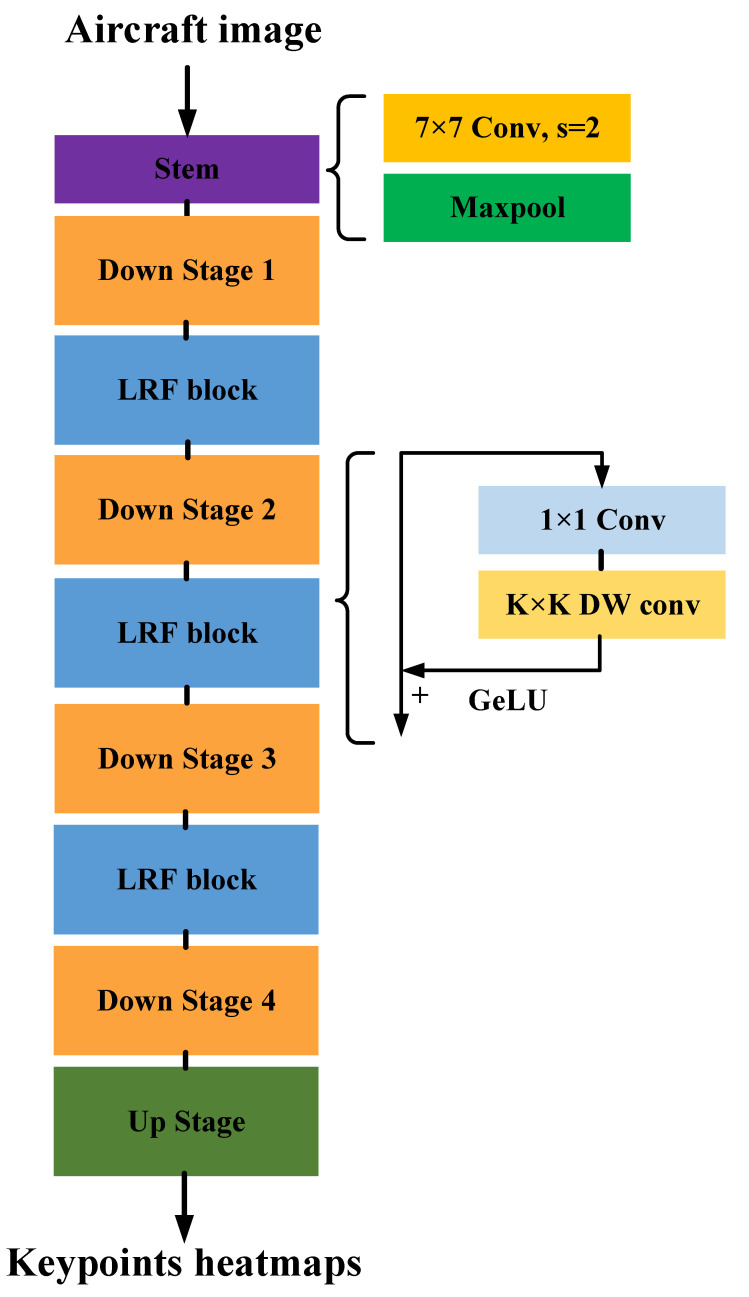
Aircraft PoseNet.

Despite Aircraft SRNet helping us recover high-resolution aircraft images, upsampling interpolation is unable to stop the loss of high-frequency information, which softens the features of the aircraft texture in high-resolution images. It is easier to recognize the geometric keypoints of the aircraft if concentrating on its overall structure rather than its texture. We suggest using a large receptive field block (LRF block) to enable CNN to broaden its field of perception and perceive the aircraft as a whole. The LRF block performs the receptive field expansion at each stage of downsampling in our aircraft PoseNet, as shown in Figure 6.

#### Large Receptive Field Block

**Figure 7:**
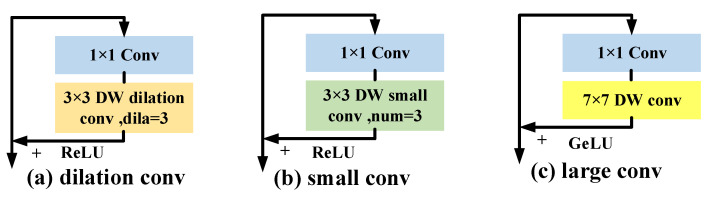
Different ways to expand the receptive field of CNN. DW conv means depth convolution.

The global structure of the aircraft is better captured by the expanded CNN receptive field. Equation (7) is used to calculate the perceptual field:

(7) $$R_{t}=R_{t-1}+\left[\left(k_{t}-1\right)*\prod_{i=1}^{t-1}{\;s}_{i}\right]$$

where the convolutional receptive field size at the *t* stage is indicated by the variable $R_{t}$. We discuss 7×7 as an example, as shown in Figure 7. The scalable receptive field of three tiny convolutions with a series of 3×3 and an atrous convolution with a dilation rate of 3 is comparable to that of a convolution with 7×7, as can be seen. However, their effective receptive fields (ERFs) [34] are actually dissimilar, as the effective receptive field Equation (8) demonstrates:

(8) $$\mathrm{ERF}∼O(k\sqrt{n})$$

The ERFs of CNN is proportional to the convolutional kernel (*k*) and proportional to the square root of the network depth (*n*), as can be seen from the relationship above. This means that expanding the network depth, which expands the ERFs of many small convolutions placed in series, is less effective than expanding the convolution kernel size. Second, the aircraft-by-pixel prediction task is impacted by the negative effects, since the atrous convolution kernel is discontinuously sampled.

ReLU has a gradient of 0 for the majority of pixels in the region away from the Gaussian kernel, as seen in Equation (1) or Figure 4, which prevents the corresponding gradients from updating and converging to the vicinity of the aircraft keypoints. According to Equation (9), GeLU causes the gradient to be continuously close to 0, which aids in the heatmap’s convergence toward the location of the aircraft keypoint.

(9) $$GELU\left(x\right)=x\cdot \frac{1}{2}\left[1+\mathrm{erf}\left(x/\sqrt{2}\right)\right]$$

where *x* represents the pixel points in the feature map, and erf(x) denotes the Gaussian error function, $\mathrm{erf}\left(x\right)=\frac{2}{\sqrt{\pi }}\int_{0}^{x}e^{-y^{2}}dy$.

## 4. Experiments

### 4.1. Aircraft Dataset

**Figure 8:**
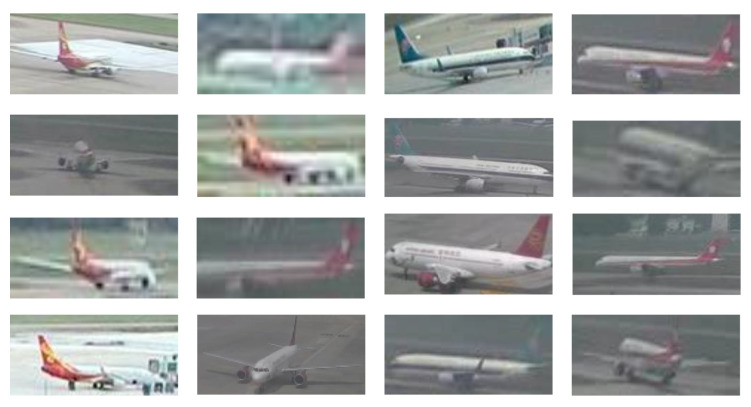
Airport surface surveillance dataset.

The Airport surface surveillance dataset, which comprises a total of over 10,000 images and incorporates images taken from public surveillance footage of civilian airport scenarios, as well as video from dozens of camera spots, includes more than 30,000 different aircraft. The majority of the pictures were taken at an oblique upper angle. This dataset, as seen in Figure 8, is thought to be difficult to analyze, since it contains a lot of blurry target images. We labeled the aircraft pose to include nine important joints with defined boundaries and a center point where the wings intersect the fuselage, as shown in Figure 2, in order to precisely characterize the construction of the aircraft.

### 4.2. Evaluation Metric

The position of the aircraft’s geometric keypoint determines the skeleton; thus, we use the widely accepted COCO evaluation criteria [32] to evaluate the aircraft pose: the Object KeyPoint Similarity index (OKS):$OKS=\frac{\Sigma_{i}\exp\left(-d_{i}^{2}/2s^{2}k_{i}^{2}\right)\delta \left(v_{i}>0\right)}{\Sigma_{i}\delta \left(v_{i}>0\right)}$. The Euclidean distance in this case between each related ground truth and the identified keypoint is $d_{i}$. The ground truth’s visibility flag is represented by the variable $v_{i}$, and the bounding box area at the object scale is represented by the variable *s*. The constant $k_{i}$ regulates the decay at each keypoint. The primary competitive indicator and metric is the average accuracy (AP) utilizing 10 OKS levels.

### 4.3. Implement Details

All experiments are executed on a GIGABYTE 3090 Ti GPU with the Faster-RCNN [35] aircraft detector in the Ubuntu 18.04, Pytorch [36] environment in order to fairly assess the superiority of the proposed method in low-quality airport field aircraft pose estimation tasks. We adhered to the standard data augmentation and training strategies used in all ablation studies to maintain experiment homogeneity and avoid CNN overfitting. Specifically, by randomly scaling (±30%), randomly rotating ($\pm 40^{\circ }$), and randomly flipping horizontally. Our initial learning rate is 1 × 10^−3^, decreasing to 1 × 10^−4^ and 1 × 10^−5^ in the 90th and 130th epochs, respectively. A total of 150 epochs. Mini-batch = 128. Using the Adam [37] optimizer, the momentum is 0.9. The following discussion assumes an input low-resolution image size of 128×96.

### 4.4. Comparison with State-of-the-Art Baseline Methods

To evaluate the effectiveness of our proposed method, we compared our method with two state-of-the-art baseline methods, namely heatmap-based methods (SimpleBaseline [38] and HRNet [39]), under a low-resolution realistic airport surface surveillance dataset. The same resolution of 128×96 is used for both model training and testing.

Table 1 demonstrates that, compared with other state-of-the-art baseline approaches, our method, which uses HRNet-W48 [39] as the foundation of the Aircraft PoseNet, achieves an AP of 89.7%. (i) With ResNet-50 serving as the foundation, SR and LRF achieve AP increases of 2% (85.3–83.3) and 3% (86.3–83.3), respectively, above Baseline. (ii) Using ResNet-101 as the backbone, SR and LRF experience AP gains of 0.7% (85.5–84.8) and 1.7% (86.5–84.8) in comparison with Baseline, respectively. (iii) SR and LRF obtain AP increases of 2.4% (87.0–84.6) and 2.1% (86.7–84.6), respectively, based on HRNet-W32. (iv) SR and LRF acquire 3.8% (89.4–85.6) and 1.3% (86.9–85.6) AP gains, respectively, based on HRNet-W48.

**Figure 9:**
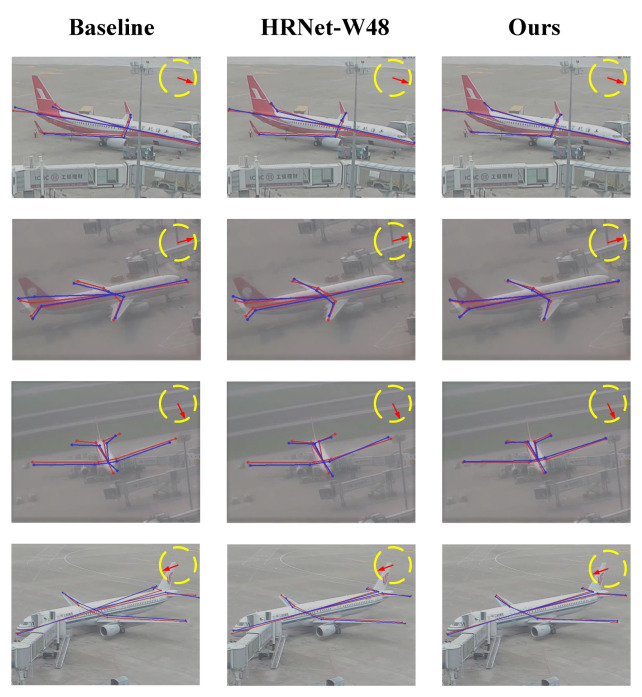
The results (blue) of the aircraft pose visualization and ground truth skeleton (red) for a better view of the aircraft skeleton.

Based on Baseline, we discovered that the SRNet preprocessing is much worse than the LRF impact. On the other hand, when HRNet is employed as the backbone of Aircraft PoseNet, the SRNet result is noticeably better than LRF. The two examples above demonstrate that while high-resolution features appear to be more significant in the case of HRNet multibranch multiscale information interaction, the gain of receptive field expansion in a single-branch architecture is more sensitive. Figure 9 displays the Baseline (ResNet-101), HRNet-W48, and our aircraft pose findings to more clearly demonstrate the efficacy of our method. The degree of overlap visually reflects the validity of the methods.

### 4.5. Ablation Studies

In this section, we first perform ablation studies on the impact of different super-resolution subnetworks on the aircraft pose estimation task to demonstrate the advantages of our method. Table 2 shows that our super-resolution subnetwork achieves the best results with relatively low GFLOPs (+3.77), significantly better than FSRCNN [41], RDN [42], and SOF-VSR [43]. Our method improves AP by 1.2% with little more computational costs than FeNet [44], which is a lightweight SR network. Then, we investigated how various receptive field expansion methods affect how well aircraft pose work is estimated. The several methods for enlarging the 7×7 receptive field are listed in Table 3, including a 3×3 atrous convolution with a dilation rate = 3, three tandem 3×3 small convolutions, and a 7×7 large convolution. The findings demonstrate that the atrous convolution is sampled in a discontinuous manner in a detrimental way for the work on estimating aircraft pose. When compared with using a single large convolution kernel directly, the tandem small convolution has a lower paradigm efficiency.

Then, we examine how different activation functions and convolutional kernel sizes affect the LRF. As shown in Table 4, we perform ablation tests on two alternative types of the backbone with different convolutional kernel sizes and different activation functions. The outcomes demonstrate that the GeLU activation function and the 11-bit convolution kernel size are the best options for this task.

## 5. Conclusions

In this paper, we propose a novel end-to-end 2D aircraft pose estimation approach to deal with the issue of aircraft pose estimation on airport surface at low resolution. The method uses a subnetwork SRNet to recover high-resolution details of the aircraft, as well as a core component LRF block to focus on the aircraft as a whole and overcome the local texture feature of the SRNet’s blurring. Through extensive experiments using the airport surface surveillance dataset, we establish in this study the necessity for high-resolution reconstruction of the low-resolution aircraft pose estimate problem. We also demonstrate the potential of a large convolutional extended receptive field. Finally, ablation studies show that diverse PoseNet methods do not all benefit equally from resolution and receptive field. Compared with the other most widely used baseline methods, our suggested method is more precise and efficient.

## Figures and Tables

**Table 1:** Comparison with state-of-the-art baseline methods.

Method	Backbone	Parameters	AP	AP${}_{M}$	AP${}_{L}$	AR	AR${}_{M}$	AR${}_{L}$
TokenPose [40]	Transformer	13.5M	78.4	80.6	83.8	83.1	81.2	89.7
Baseline [38]	Resnet-50	34.0M	83.3	82.7	86.9	88.4	83.4	94.1
Baseline+SR	ResNet-50	35.9M	85.3	85.7	87.6	90.2	86.2	**94.8**
Baseline+LRF	ResNet-50	34.4M	86.3	84.9	**89.4**	**91.2**	88.7	93.9
Baseline+both	ResNet-50	36.3M	**87.5**	**88.1**	89.3	90.9	**89.0**	93.0
Baseline [38]	ResNet-101	53.0M	84.8	81.4	**89.2**	89.4	86.2	93.0
Baseline+SR	ResNet-101	54.9M	85.5	85.5	88.0	89.7	86.3	93.4
Baseline+LRF	ResNet-101	53.4M	86.5	88.0	87.6	90.9	89.6	92.3
Baseline+both	ResNet-101	55.3M	**87.9**	**89.4**	88.6	**92.2**	**90.1**	**94.6**
HRNet-W32 [39]	HRNet-W32	28.5M	84.6	83.0	88.6	89.4	85.4	94.0
HRNet-W32+SR	HRNet-W32	30.4M	87.0	86.9	88.4	91.4	89.9	93.0
HRNet-W32+LRF	HRNet-W32	28.6M	86.7	82.7	**91.0**	91.1	88.2	**94.3**
HRNet-W32+both	HRNet-W32	30.5M	**88.7**	**91.6**	87.6	**92.3**	**91.9**	92.6
HRNet-W48 [39]	HRNet-W48	63.6M	85.6	86.7	87.3	90.2	87.7	93.1
HRNet-W48+SR	HRNet-W48	65.5M	89.4	**86.7**	92.2	92.9	91.2	94.8
HRNet-W48+LRF	HRNet-W48	63.7M	86.9	86.4	90.5	90.9	86.7	95.7
HRNet-W48+both	HRNet-W48	65.6M	**90.1**	86.6	**96.4**	**94.1**	**91.3**	**97.3**

**Table 2:** Effect of different super-resolution subnetworks on aircraft pose estimation.

Method	Backbone	GFLOPs	AP	AP${}_{M}$	AP${}_{L}$	AR	AR${}_{M}$	AR${}_{L}$
+FSRCNN [41]	ResNet-50	+4.18	83.6	84.8	84.4	89.0	85.6	90.2
+RDN [42]	ResNet-50	+16.3	84.2	85.4	85.0	89.3	86.2	91.6
+SOF-VSR [43]	ResNet-50	+15.6	84.8	85.7	86.3	89.5	86.3	92.8
+FeNet [44]	ResNet-50	+1.87	84.1	84.9	85.6	89.2	85.8	91.5
+SR	ResNet-50	+3.77	**85.3**	**86.8**	**87.6**	**90.2**	**87.3**	**94.8**
+FSRCNN [41]	HRNet-W32	+4.18	85.3	85.4	86.0	89.2	87.3	89.6
+RDN [42]	HRNet-W32	+16.3	85.9	86.5	86.2	90.0	89.9	90.1
+SOF-VSR [43]	HRNet-W32	+15.6	86.1	86.9	84.6	90.0	89.6	90.4
+FeNet [44]	HRNet-W32	+1.87	85.6	86.0	85.6	89.9	86.8	89.8
+SR	HRNet-W32	+3.77	**87.0**	**89.2**	**88.4**	**91.4**	**89.9**	**93.0**

**Table 3:** Effect of different ways of extending the reception field on aircraft pose estimation.

Conv Type	Backbone	AP	AP${}_{M}$	AP${}_{L}$	AR	AR${}_{M}$	AR${}_{L}$
Dilation	ResNet-50	82.6	81.4	85.2	88.6	85.2	92.4
Small	ResNet-50	83.8	82.9	87.0	88.8	85.5	92.5
Large	ResNet-50	**86.3**	**84.9**	**89.4**	**91.2**	**88.7**	**93.9**
Dilation	ResNet-101	84.0	85.2	85.9	89.5	86.3	93.1
Small	ResNet-101	85.3	86.3	87.4	90.0	87.0	**93.3**
Large	ResNet-101	**86.5**	**88.0**	**87.6**	**90.9**	**89.6**	92.3
Dilation	HRNet-W32	82.1	79.5	85.0	87.0	84.5	89.8
Small	HRNet-W32	85.1	80.7	90.0	89.6	85.7	94.1
Large	HRNet-W32	**86.7**	**82.7**	**91.0**	**91.1**	**88.2**	**94.3**
Dilation	HRNet-W48	84.8	81.4	89.2	89.4	86.2	93.0
Small	HRNet-W48	85.9	83.0	89.3	90.9	**88.5**	93.6
Large	HRNet-W48	**86.9**	**86.4**	**90.5**	**90.9**	86.7	**95.7**

**Table 4:** Effect of different sizes of reception field on aircraft pose estimation.

Kernel	Backbone	Activation Function	AP	AP${}_{M}$	AP${}_{L}$	AR	AR${}_{M}$	AR${}_{L}$
3	ResNet-50	GeLU	83.6	84.4	85.2	88.7	85.9	91.9
7	ResNet-50	GeLU	**86.3**	**84.9**	89.4	**91.2**	**88.7**	93.9
11	ResNet-50	GeLU	85.8	84.4	90.3	89.3	85.1	**94.2**
13	ResNet-50	GeLU	84.9	79.5	**91.6**	88.7	84.0	94.1
7	ResNet-50	ReLU	84.0	83.4	88.0	88.3	83.7	93.5
3	HRNet-W32	GeLU	84.8	**87.4**	82.3	89.6	**91.1**	87.9
7	HRNet-W32	GeLU	**86.7**	82.7	91.0	**91.1**	88.2	**94.3**
11	HRNet-W32	GeLU	86.6	82.7	**91.3**	91.1	88.2	94.3
13	HRNet-W32	GeLU	85.8	84.4	90.3	89.3	85.1	94.2
7	HRNet-W32	ReLU	86.5	83.6	90.4	90.9	89.0	93.0

## Data Availability

The data used to support the findings of this study are available from the corresponding author upon request.

## Abbreviations

The following abbreviations are used in this manuscript:

MDPI	Multidisciplinary Digital Publishing Institute
DOAJ	Directory of open access journals
TLA	Three letter acronym
LD	Linear dichroism
